# Voriconazole-Induced Periostitis: A Case Report

**DOI:** 10.7759/cureus.45947

**Published:** 2023-09-25

**Authors:** Tristan D Cooper-Roth, Caleb R Boehler, Stefano J Natali, Sami R Ahmed, Tyson S Chadaz

**Affiliations:** 1 Radiology, University of Arizona College of Medicine-Tucson, Tucson, USA

**Keywords:** voriconazole toxicity, msk radiology, musculoskeletal radiology, periostitis, voriconazole therapy

## Abstract

Voriconazole-induced periostitis (VIP) is an uncommon side effect typically seen in immunosuppressed patients requiring prolonged antifungal therapy. These patients can present with bone pain, fragility, and elevated alkaline phosphatase (ALP). We present a case of VIP in a 72-year-old immunocompromised female on antifungal therapy presenting with a comminuted intertrochanteric fracture after a ground-level fall. VIP, although rare, should be included as a differential diagnosis for patients presenting with bone pain and/or fractures with radiographic features of periostitis. This is particularly true when there is a history of or prior imaging suggesting a solid organ transplant. In these cases, a dedicated review of current medications noting long-term voriconazole use in the absence of underlying rheumatologic disease can result in a confident diagnosis.

## Introduction

Voriconazole is a second-generation broad-spectrum triazole antifungal medication that is primarily used as a first-line agent in the treatment of invasive *Aspergillosis* and *Candida albicans*. Invasive aspergillosis is a life-threatening fungal infection that primarily affects patients who are immunocompromised (e.g., bone marrow or organ transplant recipients, hematologic malignancies, acquired immunodeficiency syndrome). One of the uncommonly seen daily/cumulative dose-related side effects of voriconazole is voriconazole-induced periostitis. Patients with voriconazole-induced periostitis can demonstrate bone pain/fragility, elevated plasma alkaline phosphatase (ALP), as well as radiological imaging findings of periostitis in the absence of rheumatologic disease [1]. These radiological features can be observed on plain radiography as multifocal periostitis with periosteal thickening/reaction with new brittle bone formation. Here, we present a case of a 72-year-old female with suspected voriconazole-induced periostitis. 

## Case presentation

**Figure 1:**
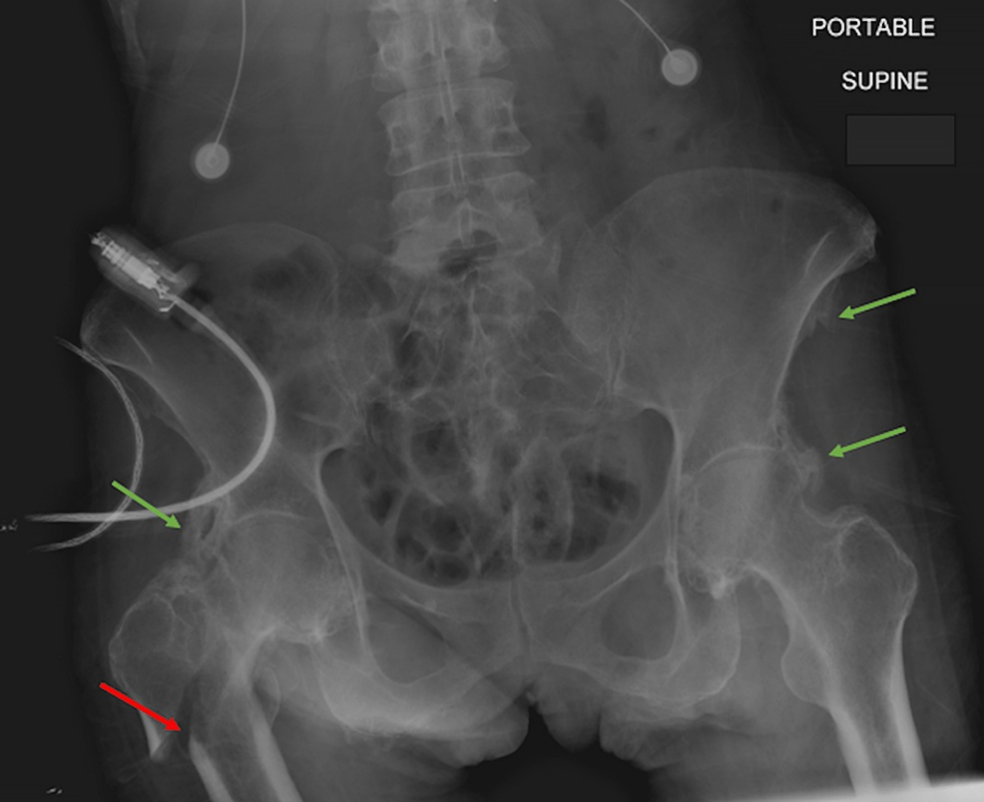
AP supine pelvic radiograph at the time of patient presentation for ground-level fall, demonstrating the primary finding of a comminuted intertrochanteric fracture of the right hip (red arrow) and the incidental finding of extensive periostitis and enthesopathy of the bilateral attachment sites of the rectus femoris and sartorius (green arrows). Image date for reference: late 2022.

**Figure 2:**
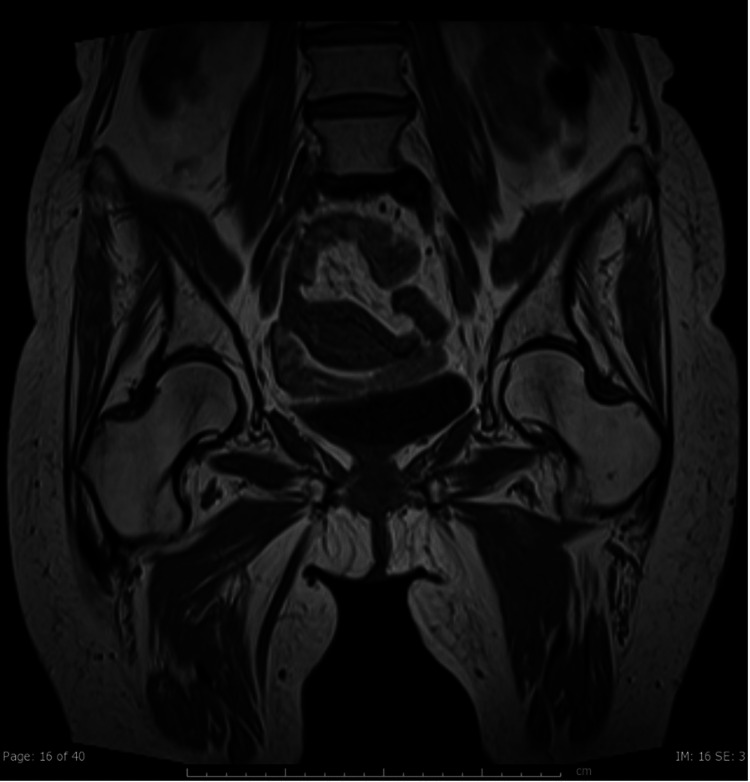
Coronal T1 MRI of the bilateral lower extremities two years before presentation, demonstrating that the abnormalities seen on Figure 1 are an interval development after starting voriconazole. Image date for reference: mid-2020.

**Figure 3:**
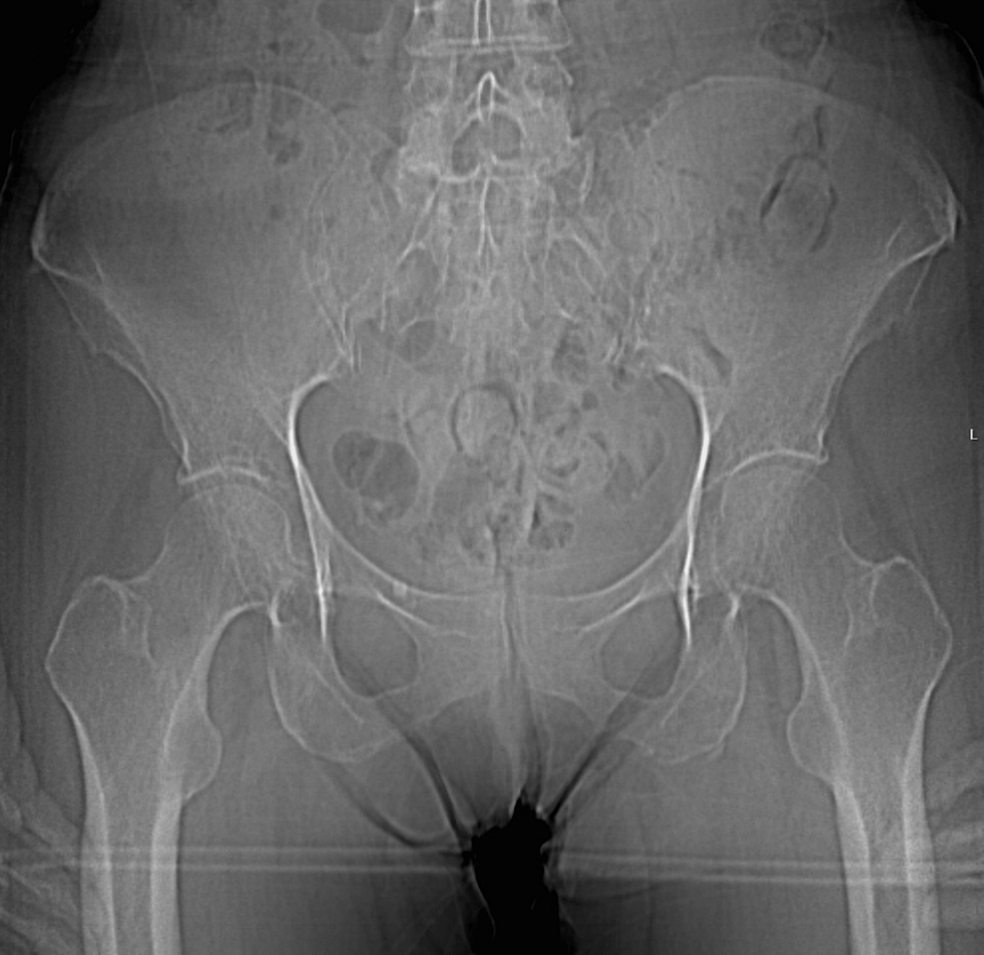
AP supine pelvic radiograph three years before presentation, demonstrating that the abnormalities seen on Figure 1 are an interval development after starting voriconazole. Image date for reference: late 2019.

**Figure 4:**
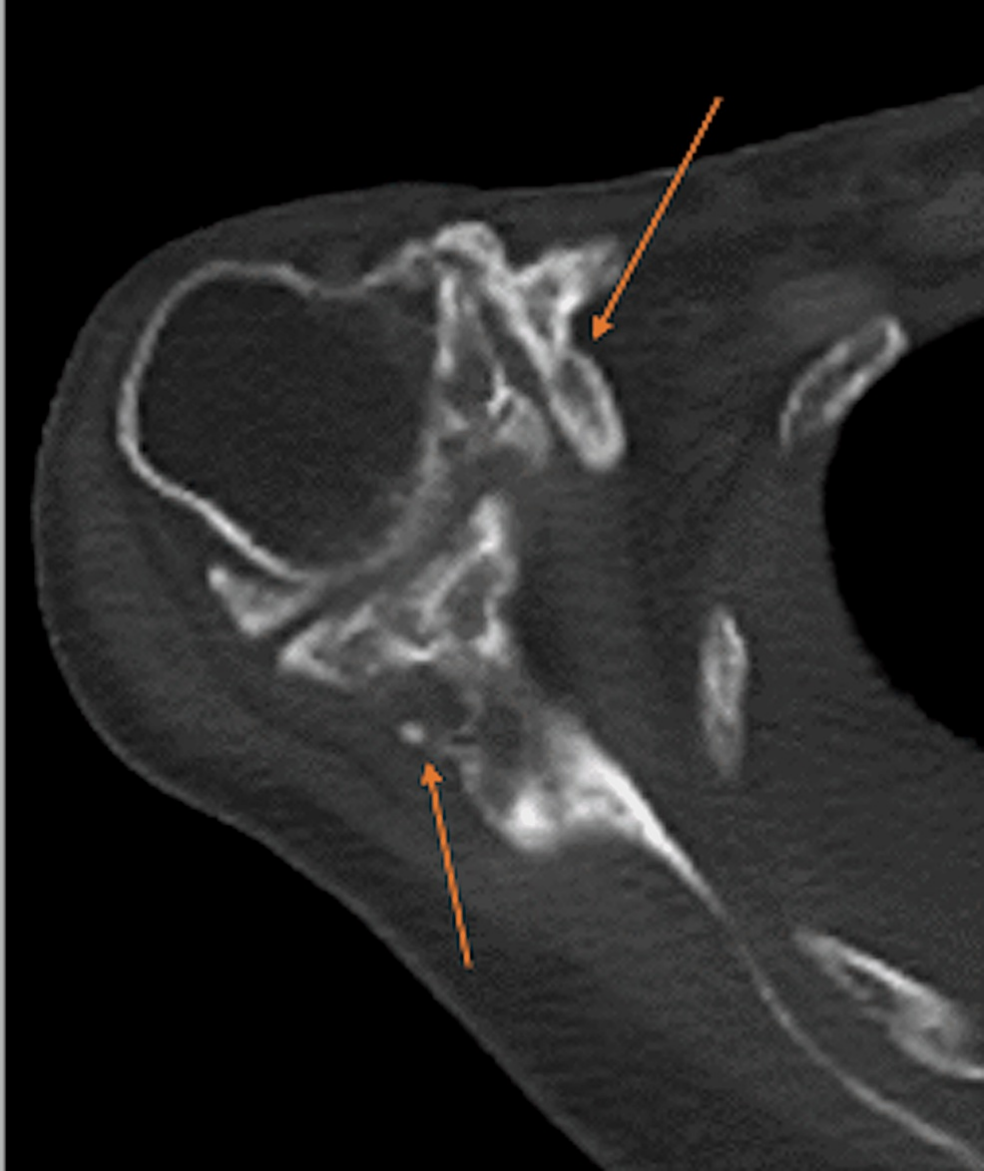
An axial CT image of the right shoulder shows extensive periostitis and large osteophyte formation (orange arrows) about the right glenohumeral joint, with associated impingement about the shoulder in all directions. Image date for reference: late 2022.

**Figure 5:**
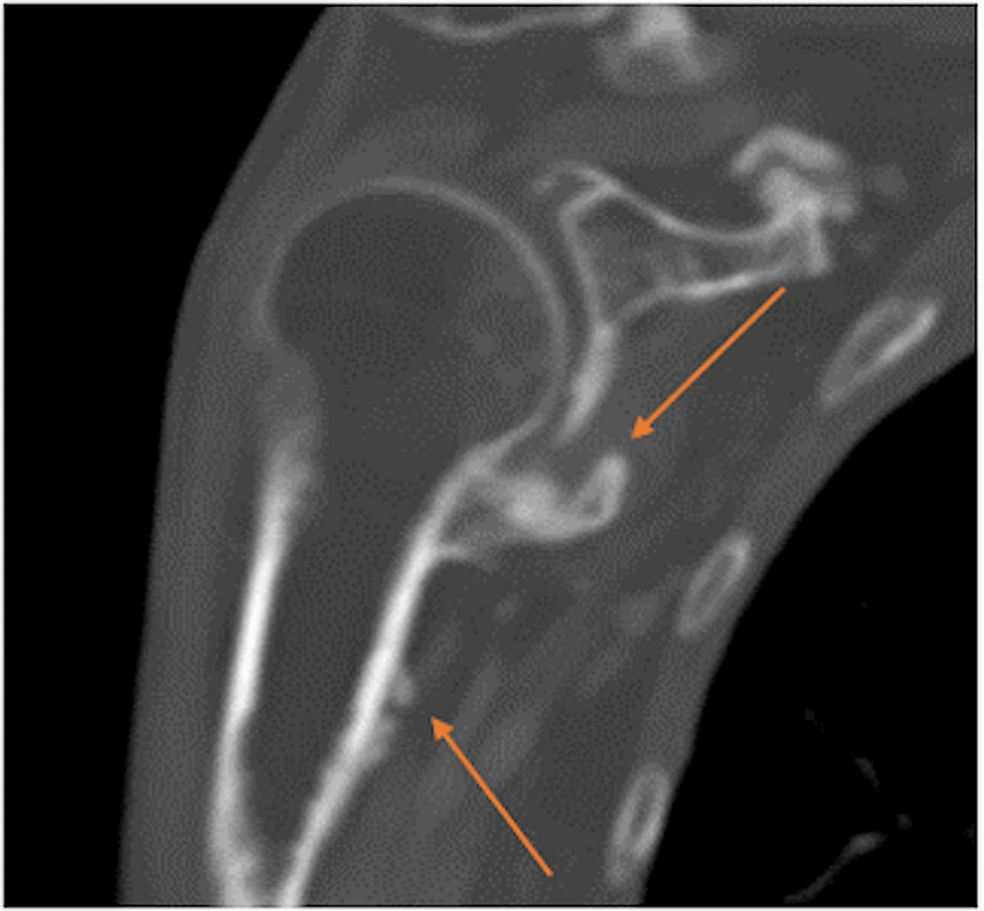
A coronal CT image of the right shoulder shows extensive periostitis and large osteophyte formation (orange arrows) about the right glenohumeral joint, with associated impingement about the shoulder in all directions. Image date for reference: late 2022.

**Figure 6:**
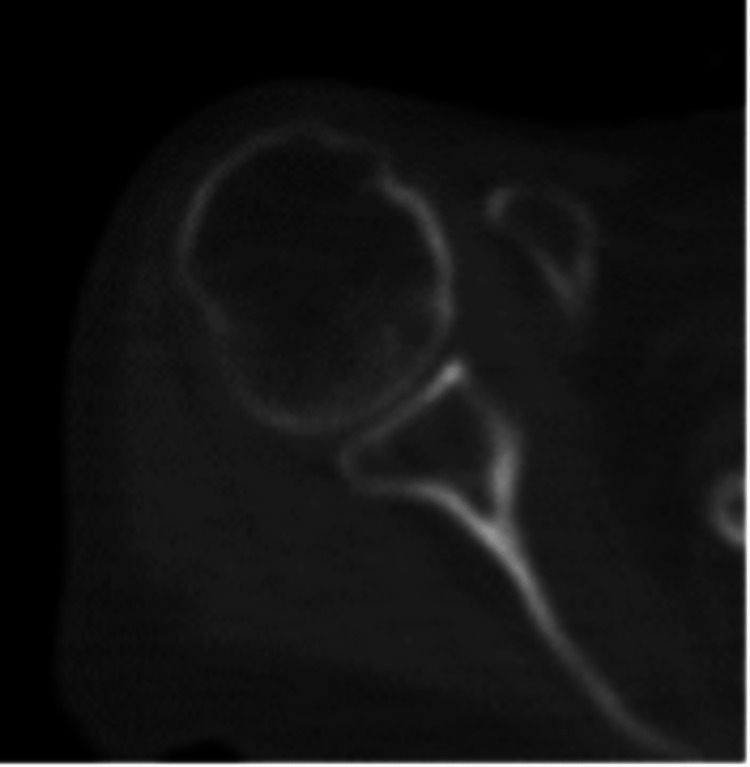
An axial CT image of the right shoulder three years ago demonstrated a glenohumeral joint without osteophyte formation or periostitis. Image date for reference: mid-2019

A 72-year-old female presented to the emergency department for the evaluation of right hip pain after a ground-level mechanical fall. A comminuted right intertrochanteric fracture was noted on an AP supine pelvic radiograph. The patient’s past medical history was significant for non-Hodgkin’s lymphoma status post-allogenic stem cell transplant, complicated by chronic graft versus host disease and invasive aspergillosis of the brain and lungs. The patient had been on long-term, high-dose voriconazole (200-300 mg BID; start date three years before her hip fracture presentation). Additional findings on the AP supine pelvic radiograph demonstrated an interval change of extensive periostitis of the bilateral attachment sites of the rectus femoris and sartorius (Figure 1), which were new findings when compared to an MRI of the bilateral lower extremities and pelvic radiograph dated approximately two to three years prior (Figures 2, 3). Additionally, the interval evolution of periostitis and osteophyte formation can be seen on CT scans of the right upper extremity that were performed three years apart (Figures 4-6). The patient's lab work was significant for an ALP of 577 international units per liter (IU/L; normal range: 44-147 IU/L). 

## Discussion

Triazole antifungal agents contain varying amounts of fluoride within their chemical structure, with voriconazole containing three fluoride atoms. A standard 400-mg dose of voriconazole contains approximately 60 times the amount consumed in municipal tap water, which is typically 1 mg per liter [2]. Voriconazole is metabolized primarily via the hepatic cytochrome P450 and CYP2C19 pathways, with approximately 42% of the drug remaining unbound by plasma proteins and 5% metabolized to free fluoride, elevating unbound serum fluoride levels [3,1]. The absorption of excess fluoride within the extracellular bony matrix may alter its normal crystal structure to form fluorapatite; excess deposits of such are deemed toxic to periosteal bone [2]. 

Unlike normal calcium hydroxyapatite, fluorapatite induces a disorganized osteoblastic reaction, promoting excess bone mineralization, and is believed to be one of the primary mechanisms of periosteal reaction in patients taking voriconazole [3]. Continued exposure to fluorapatite may lead to characteristic imaging features, including periostitis, osteosclerosis, enthesopathic changes, and eventually osteoporosis, with the constellation of findings termed skeletal fluorosis [3,4]. The descriptive terminology attributed to the periostitis pattern includes fluffy, feathery, and intermittently nodular morphology with a primarily asymmetrical distribution [4]. 

Common clinical manifestations of voriconazole-induced periostitis typically develop within six months to three years following the initiation of therapy [1]. These manifestations include diffuse bone pain, dental fluorosis, and an increased susceptibility to fractures, given the disorganized, brittle bony architecture [2]. Multiple skeletal sites are typically involved, with the ribs, forearms, lower extremities, and shoulders being the most common [1]. In conjunction with the previously described characteristic imaging features, serology will demonstrate elevated alkaline phosphatase and elevated serum fluoride levels, more commonly when periostitis is present [1]. 

There are various pathologies that may cause diffuse or focal periostitis, which should be further investigated if VIP is to be ruled out as the primary diagnosis. Differential diagnoses for periostitis include hypertrophic osteoarthropathy, thyroid acropachy, and hypervitaminosis A [5]. Additionally, periostitis can be seen in the setting of inflammatory arthropathy (psoriatic arthritis), post-traumatic healing processes, infection (osteomyelitis), and multiple benign and neoplastic lesions of the bone. Periosteal reaction alone is a nonspecific radiographic finding; however, and in many instances, correlation with the clinical presentation must be made by the primary provider. The aforementioned differential diagnoses were felt to be less likely to be the cause of periostitis, as the clinical workup and patient presentation did not favor these etiologies.

## Conclusions

Voriconazole-induced periostitis is a musculoskeletal side effect that should be considered in patients with long-term voriconazole treatment presenting with elevated ALP, bone pain/fracture and/or radiologic findings of periostitis in the absence of rheumatologic disease. Although this entity is uncommonly seen, we hope that this case report provides insight and increased awareness to clinicians, as this side effect should be included as a differential diagnosis in this patient population. 
